# Unnecessary test ordering in clinical trials: human chorionic gonadotropin as an example

**DOI:** 10.1515/almed-2019-0004

**Published:** 2020-01-07

**Authors:** Estibaliz Alegre, Miguel F. Sanmamed, Teresa Sendino, Jose Luis Perez-Gracia

**Affiliations:** Biochemistry Laboratory, Clínica Universidad de Navarra, Pamplona, Spain; Oncology Department, Clínica Universidad de Navarra, Pamplona, Spain; Navarra Institute for Health Research (IdiSNA), Pamplona, Spain; Centro de Investigación Biomédica en Red de Cáncer (CIBERONC), Madrid, Spain

**Keywords:** clinical trial, chorionic gonadotropin (hCG)

To the Editor,

Among the requisites to be included in a clinical trial, patients have to undergo a panel of analytical tests. These tests are mandatory and are usually requested systematically to all patients, regardless of their clinical situation. Because some of these tests are, in fact, exclusion criteria, their indiscriminate request, out of their intended purpose, may have noxious consequences for patients. Besides liver and renal function tests, a test for human chorionic gonadotropin (hCG) is one of the tests most frequently requested, in order to prevent pregnant women from entering or continuing in a clinical trial.

hCG is a hormone that has been traditionally used as the pregnancy marker par excellence, with a high specificity. However, when requested without considering clinical context, it might become a confounding result and even complicate patient inclusion in a clinical trial.

An example is request for an hCG test in postmenopausal women who may present plasma hCG values discretely above the cutoff established for pregnancy detection. This elevation has been observed in our laboratory in 8.3% of women older than 45 years of age, with an hCG test requested prior to entering in a clinical trial. These slightly elevated values (<25 UI/L) may be explained by a pituitary hCG production, and higher cutoffs can be established for postmenopausal women to take this into account [1]. Nevertheless, additional follicle-stimulating hormone (FSH) and luteinizing hormone (LH) tests could also be needed to confirm the postmenopausal status [2].

Another reason for hCG increase can be its ectopic production by different types of tumor. In those cases, hCG levels are higher than those observed in postmenopausal women (above 100 UI/L). As in the previous situation, additional tests have to be performed to avoid patient exclusion from the clinical trial as a consequence of a nonexistent pregnancy. This situation can be even more confusing as LH and FSH tests can be eventually useless if the woman is not postmenopausal. In those cases, it is worthy to check if elevated hCG levels are in fact due to high levels of the free beta subunit of hCG because some of the commercial kits available for hCG measurement detect not only the complete heterodimer hCG but also the free beta subunit. This verification is useful because it has been described that some epithelial cancers are responsible for an ectopic free beta subunit production [3], [4].

In both situations, elevated hCG levels might not provoke an ultimate exclusion of patients from the clinical trial, but they do imply a delay in the inclusion process because of the additional tests required to clarify the origin of those hCG levels. That delay could have been prevented if initial hCG tests were not requested and/or performed in women in whom a pregnancy is not possible, usually due to the age of the patients.

Some authors have already questioned the use of hCG as an exclusion criterion for clinical studies [5]. In fact, most of the current protocols usually do not indicate the need for an hCG test itself but that the clinician rules out the possibility of a pregnancy instead. This can be achieved by means of hCG, but also by considering the age of the patient and/or other circumstances such as a previous hysterectomy. Consequently, an indiscriminate request of hCG tests from clinical trials units is not always justified.

In conclusion, a proactive attitude should be encouraged in laboratory analysts in terms of discussing with the requesting physicians the convenience of performing hCG tests in certain patients. A clinical trial should not always justify the request of certain tests that could be misleading or even absurd in the clinical context. Fluent communication between both analysts and physicians would reduce the number of unnecessary tests and the potential inconveniences associated with them.
